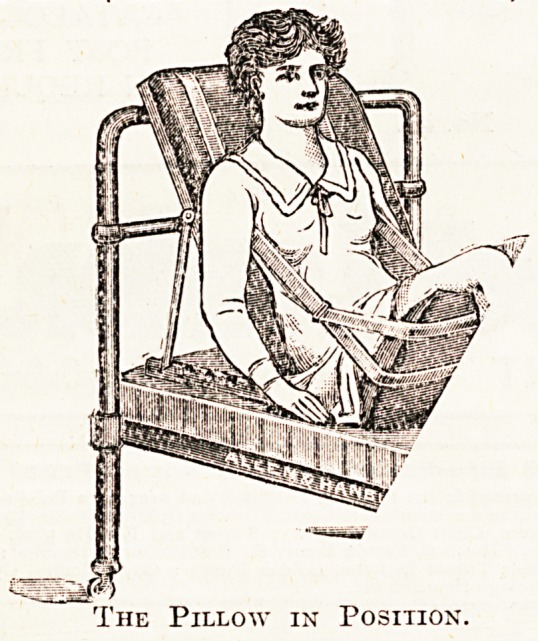# Institutional Needs

**Published:** 1913-05-24

**Authors:** 


### Institutional Needs.

### PILLOW AND STRAPS FOR MAINTAINING THE "FOWLER POSITION."

"A trial extending over three years," writes Mr. C. Hamilton Whiteford, of 5 Sussex Terrace, Plymouth, "having demonstrated the comfort of these straps to both patient and nurse, Messrs. Allen and Hanburys have now, at my request, undertaken their manufacture. The pillow is 2 ft. in length and 1 ft. in width, is stuffed firmly, and is covered with mackintosh. In the centre of one side a semicircle is cut. Two webbing straps are attached to one face of the pillow in its long axis; the adjacent ends of these straps converge to terminate in a buckle at each end of the pillow. Two separate straps, having at one end a leather loop, connect at this end with the head rail of the bed, and at the other end with the buckles on the pillow. The pillow and straps are devised for maintaining, not for dragging the patient into, the Fowler position. The patient is supported, at the required angle, on a bed-rest. The two loose straps are looped to the top rail at the head of the bed, about 2 ft.

The Pillow in Position.

apart. (Note.—If the head rail of the bed is on a lower level than the patient's shoulders, the straps must pass from the bed-rail up over the top of the bed-rest and then under or outside the patient's arms down to meet the buckles.) The pillow with its straps below, *i.e.*, next the bed, and the edge with the semicircular gap upwards, *i.e.*, next the rectum, is covered with a warmed towel and slid across the bed beneath the thighs, reaching up to the buttocks. The ends of the straps from the head of the bed are passed through the buckles of the pillow. A nurse on each side of the patient, with one hand under the pillow, first gradually raises the pillow and thighs, and then, with the other hand, tightens the straps in the buckles. These straps must be tightened equally until they are quite taut. On removing the hands from beneath the pillow, the patient is supported on the seat of a swing, but the seat of the swing, *i.e.*, the pillow, rests, not against the buttocks, but against the back of the thighs, immediately in front of the buttocks. The soles of the feet are supported on an ordinary pillow. The semicircular opening, lying opposite the perineum, allows access to the urethra and rectum, and is especially useful if saline solution is being administered per rectum."

### HYDROPYRIN AND KALMOPYRIN.

The popularity of acetyl-salicylic acid continues not only among medical men, but also among the large army of self-drugging amateurs who so uniformly drift eventually to the doctor. It was only to be expected that such a useful drug would be very soon put upon the market in combination with well-tried bases; this has now been done by Messrs. Alfred Bishop, Ltd., of 28 Spelman Street, E.C. The two salts now on the market under the above names are those of lithium and calcium. These two salts have not only the inherent therapeutic advantages due to the presence of the bases, but there is also to be considered the fact that they are both very readily soluble in water. This advantage over the acetyl-salicylic acid in its familiar isolation is so obvious that in itself this quality must necessarily make for the immediate popularity of these new *soluble* salts.

In therapeutic properties the two salts, Hydropyrin and Kalmopyrin, are practically identical, and the choice between them depends upon the circumstances of individual cases. Hydropyrin, the lithium salt of acetyl-salicylic acid, contains 3.76 per cent. lithium, with 96.24 per cent. acetyl-salicylic acid, whilst Kalmopyrin contains 10 per cent. calcium, with 90 per cent. acetyl-salicylic acid. The indications for the use of these salts are practically the same as for acetyl-salicylic acid or "aspirin," but on account of their ready solubility they should give more definite and efficient results. They are stable in solution and are easily prescribed with other drugs; for children (who naturally dislike powders) they may be given in syrups. By avoiding the use of tablets a check is placed upon self-medication.

## Figures and Tables

**Figure f1:**